# Human infections caused by *Staphylococcus argenteus* in Germany: genetic characterisation and clinical implications of novel species designation

**DOI:** 10.1007/s10096-020-03950-4

**Published:** 2020-06-23

**Authors:** Farah Alhussein, Judith Fürstenberg, Rosmarie Gaupp, Janina Eisenbeis, Katharina Last, Sören L. Becker, Cihan Papan

**Affiliations:** Centre for Infectious Diseases, Institute of Medical Microbiology and Hygiene, Saarland University, Kirrberger Strasse, Building 43, 66421 Homburg, Germany

**Keywords:** *Staphylococcus argenteus*, Novel species, Emerging pathogen, Multilocus sequence typing

## Abstract

We report a series of *Staphylococcus argenteus* infections from Saarland, Germany. Travel histories were unremarkable for extra-European sojourns, indicating an autochthonous transmission mode. Multilocus sequence typing revealed that all isolates were members of the clonal complex CC2250. In only one case, guideline-adherent treatment with an isoxazolyl penicillin was prescribed. Our report illustrates the perils of novel species designations, which may lead to misconceptions and suboptimal treatment choices among clinicians.

## Introduction

In 2015, a former member of the *Staphylococcus aureus* lineage called clonal complex (CC) 75 was delimited as an independent species called *Staphylococcus argenteus* [1]. Its identification in routine diagnostics has only recently become accurate with the update of commercial databases used for matrix-assisted laser desorption/ionization time-of-flight mass spectrometry (MALDI-TOF MS) [2, 3]. There have been conflicting reports on its pathogenicity compared to *S. aureus* [4–6]. Most data originate from Asia, Australia, and the Amazon region [6, 7], while reports from Europe are scarce [8], leaving a need for reliable epidemiological and clinical data.

## Methods

We searched our laboratory information system database for all identifications of *S. argenteus* and *S. aureus* between October 2018 and December 2019 in clinical samples (excluding surveillance screenings). All specimens were handled according to standard microbiological procedures. Isolates were identified by MALDI-TOF MS using Microflex LT mass spectrometer (Bruker Daltonics, Bremen, Germany). Antimicrobial susceptibility testing (AST) was performed using VITEK II (BioMérieux, Marcy l’Étoile, France) and interpreted according to the guidelines of the European Committee on Antimicrobial Susceptibility Testing (EUCAST, version 9.0). Multilocus sequence typing (MLST) was performed using FastStart High Fidelity PCR System (Roche, Germany) and primers as described previously [6, 9, 10]. Sequence types (ST) were assigned using the *S. aureus* MLST Database website (https://pubmlst.org/saureus/). Patients with an infection, as opposed to colonization, with *S. argenteus* were identified through the clinical microbiologist’s documentation. A chart review was performed on infected patients using the hospital information system. Written informed consent was obtained from patients for whom the clinical course is described below.

## Results

### Case descriptions

#### Patient #1

A 58-year-old male with hydrocephalus presented due to protracted wound healing adjacent to the ventriculoperitoneal (VP) shunt, which had been placed 3 months earlier. C-reactive protein (CRP) was 6.6 mg/L. Wound opening and debridement yielded purulent discharge, and the VP shunt was removed. Cultures from intraoperative wound swabs and cerebrospinal fluid (CSF) grew *S. argenteus*. Intravenous cefuroxime was initiated empirically and a new VP shunt was implanted after three consecutive, sterile CSF cultures. The patient improved quickly and was discharged after 16 days of intravenous therapy. Oral cefuroxime was maintained for another 5 days. No other infection occurred during an 8-month follow-up.

#### Patient #2

A 60-year-old male with esophageal adenocarcinoma was hospitalized because of dyspnea, malaise, and pain at the exit site of his port catheter. On physical examination, a painful redness of the port site was noted. Systolic blood pressure was 70 mmHg, quickly responding to volume resuscitation. Laboratory examination showed a CRP of 279 mg/L. Due to a suspected central-line-associated bloodstream infection (CLABSI), the port system was explanted. A wound swab and the catheter tip grew *S. argenteus*, while blood cultures remained sterile. After a 16-day course of intravenous piperacillin/tazobactam, the patient improved, and a peripherally inserted central catheter (PICC) was placed. Three months later, he presented with a painful redness of the PICC entrance site. The PICC was removed, and oral clindamycin was started. *S. argenteus* grew from the catheter tip and in blood culture, alongside *Bacillus cereus*, *Enterobacter cloacae* complex, and *Enterococcus faecalis*. The patient was started on intravenous flucloxacillin and meropenem. Follow-up blood cultures remained sterile, and a transesophageal echocardiography revealed no pathologies. After 14 days, he was discharged home in a good condition. A follow-up period of 3 months has been unremarkable since.

#### Patient #3

An 80-year-old male was referred for evaluation of pain in his right hand after previous surgery. On examination, the surgical site was unremarkable. CRP was 148 mg/L. Blood cultures were collected before starting intravenous cefuroxime and became positive with *S. argenteus*. Following rapid improvement, the patient was transferred back to the referring hospital after 3 days. The therapy was switched to flucloxacillin, according to AST results. One month later, the patient was re-admitted due to back pain and elevated inflammation markers. Magnetic resonance imaging of the spine confirmed vertebral osteomyelitis with intraspinal empyema. The patient underwent neurosurgery and escalation of antimicrobial therapy to meropenem and vancomycin. All microbiological specimens obtained during surgery remained sterile. The patient received intravenous flucloxacillin followed by oral clindamycin for 6 weeks each. No other infectious complications were noted during a follow-up of 6 months.

#### Patient #4

A 52-year-old female presented with unilateral otorrhea and decreased hearing. She suffered from chronic external otitis during the ten preceding years. The auditory canal was swollen, with yellowish secretion and scaling of the meatus. A swab from the ear grew *S. argenteus*. A 1-week course of oral clindamycin and topical ofloxacin was initiated, leading to some clinical improvement, with a long-term follow-up visit of 6 months still pending.

### Identification and characterization of *Staphylococcus argenteus* isolates

Apart from the patients described, *S. argenteus* was detected in four other patients from skin swabs (*n* = 3) and central venous catheter tip (*n* = 1), all of which were deemed as colonization. During the study period, a total number of 1013 *S. aureus* isolates were identified. Thus, the ratio of *S. argenteus* to *S. aureus* detections was 0.8%. None of the four infected patients had a recent travel history outside Europe (patient #2 reported travels to Belgium and France 2 years earlier).

**Fig. 1:**
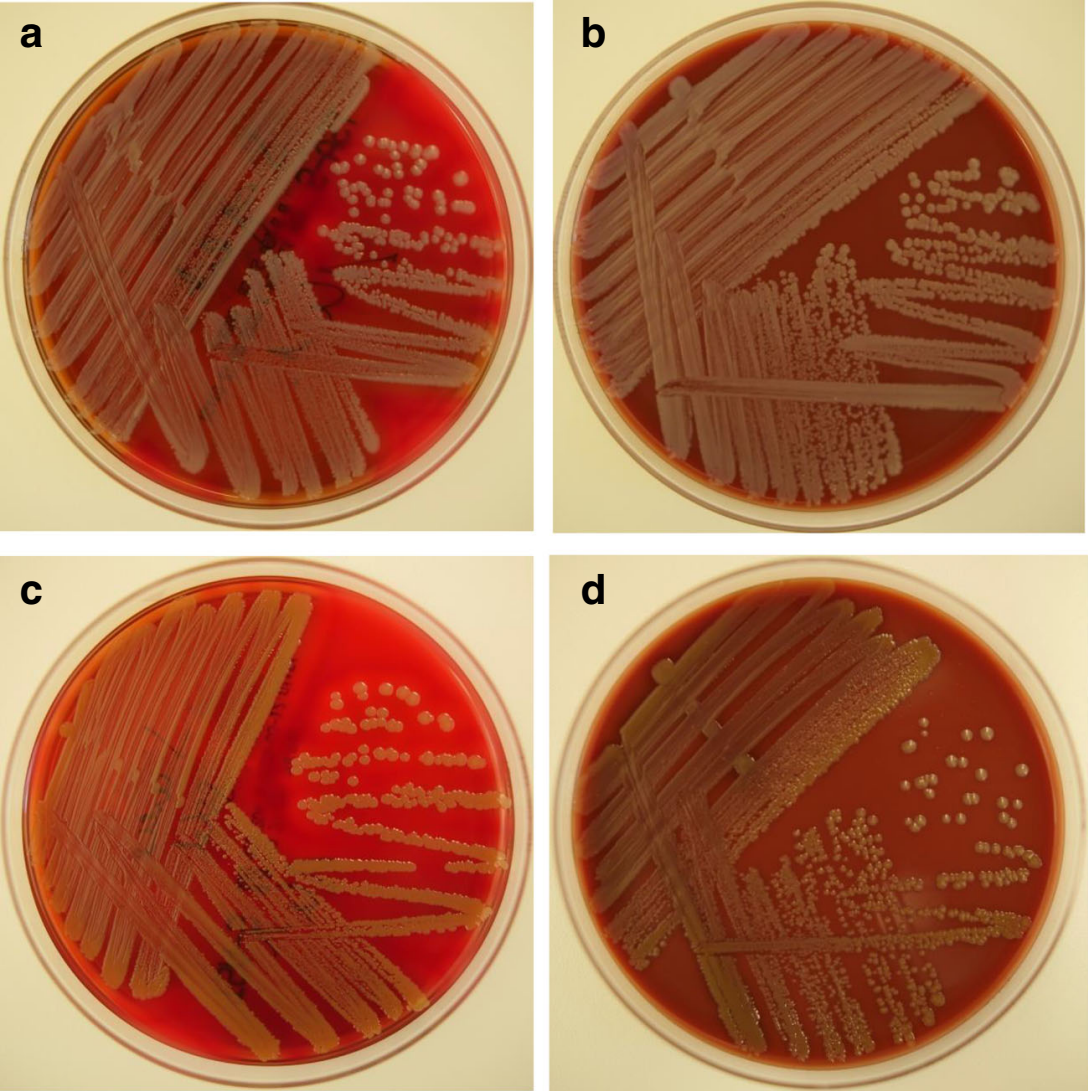
Colonies of *Staphylococcus argenteus* after 24 h of incubation at 35 °C, on blood agar (**a**) and chocolate agar (**b**), in comparison to *Staphylococcus aureus*, on blood (**c**) and chocolate agar (**d**)

On blood and chocolate agars, *S. argenteus* grew in large, white-grayish colonies with strong beta-hemolysis and a lack of pigment in comparison to *S. aureus* (Fig. 1 a–d). AST showed no methicillin-resistant isolates (Table 1).

**Table 1:** Minimal inhibitory concentrations (MIC; in mg/L) of *S. argenteus* isolates detected in clinical specimens in Homburg, Germany, between October 2018 and December 2019. MICs are given either as indicated or as smaller or equal to (≤); *Cotrimoxazole MIC 10 mg/L

Antimicrobial agent	Number of isolates with indicated MIC										Susceptible	Resistant
	0.12	0.25	0.5	1	2	4	8	16	32	64		
Penicillin G	5		3								5/8 (62.5%)	3/8 (37.5%)
Flucloxacillin		3	5								8/8 (100%)	–
Gentamicin			8								8/8 (100%)	–
Ciprofloxacin			7	1							8/8 (100%)	–
Moxifloxacin		8									8/8 (100%)	–
Erythromycin		7	1								8/8 (100%)	–
Clindamycin		8									8/8 (100%)	–
Linezolid				1	7						8/8 (100%)	–
Daptomycin		3	5								8/8 (100%)	–
Vancomycin			4	4							8/8 (100%)	–
Tigecyclin	8										8/8 (100%)	–
Fosfomycin							3	3	1	1	7/8 (87.5%)	1/8 (12.5%)
Fusidic acid			8								8/8 (100%)	–
Cotrimoxazole							8*				8/8 (100%)	–

All eight isolates were identified as belonging to CC2250 by MLST (Table 2). Specifically, seven isolates were identified as ST2250, while one isolate revealed a new *pta* allele with a G237T point mutation that was designated as a new sequence type ST5869. None of the eight *S. argenteus* isolates was tested positive for panton-valentine leucocidin, as tested by the GenoType Staphylococcus assay (Bruker-Hain-Lifescience, Germany).

**Table 2:** Primers used for MLST

Locus	Primer name	Primer sequence (5′-3′)	Reference
arcC	arcC-Up	TTGATTCACCAGCGCGTATTGTC	[9]
	arcC-Dn	AGGTATCTGCTTCAATCAGCG	
aroE	aroE745-up	TTATCACCGTCGATGCATAGTGCA	[10]
	aroE255-down	CGGAGTAGTATTTATCACAATATC	
glpF	glpF-Up	CTAGGAACTGCAATCTTAATCC	[9]
	glpF-me-r	CCGGCAATTGGTCCTAAGAT	[6]
gmk	gmk-Up	ATCGTTTTATCGGGACCATC	[9]
	gmk-me-r	AGTGCTCAGCTTCTACAATACA	[6]
pta	pta-Up	GTTAAAATCGTATTACCTGAAGG	[9]
	pta-Dn	GACCCTTTTGTTGAAAAGCTTAA	
tpi	tpi-Up	TCGTTCATTCTGAACGTCGTGAA	[9]
	tpi-Dn	TTTGCACCTTCTAACAATTGTAC	
yqiL	yqiL-Up	CAGCATACAGGACACCTATTGGC	[9]
	yqiL-Dn	CGTTGAGGAATCGATACTGGAAC	

## Conclusions

Our report on patients with *S. argenteus* infections points to a likely autochthonous acquisition in Germany, due to the lack of a recent travel history. Previous literature from high-prevalence settings indicated that *S. argenteus* accounts for 4–5% of all *S. aureus* strains detected in clinical samples [6], which is higher than in Europe (< 1%) [8, 11]. The ratio observed at our institute (0.8%) is in line with other European countries. Similarly, all of our isolates belonged to CC2250, which is among the most frequent clonal complexes [3].

Our data confirm the pathogenicity of *S. argenteus* and extend the spectrum of disease to foreign-body infections. In analogy to *S. aureus* bacteraemia, isoxazolyl-penicillins have been used to treat *S. argenteus* infections [5]. The observation that only one patient received flucloxacillin at the initial infection shows that the designation as a “different” staphylococcal species may have mislead clinicians towards a potentially suboptimal treatment, which may be associated with worse outcomes. In anticipation of such misinterpretations, the ESCMID Study Group on Staphylococci and Staphylococcal Diseases proposed to specifically add the remark “member of the *S. aureus* complex” on the microbiology report, in addition to the immediate notification of the clinician [3].

Our report contributes to the understanding of *S. argenteus* and calls for an increased awareness among microbiologists and clinicians. Further studies pertaining to *S. argenteus* should aim at elucidating its clinical relevance, epidemiology, and potential for nosocomial transmission and endogenous infections.

## References

[1] Tong SY, Schaumburg F, Ellington MJ, Corander J, Pichon B, Leendertz F (2015). Novel staphylococcal species that form part of a *Staphylococcus aureus*-related complex: the non-pigmented Staphylococcus argenteus sp. nov. and the non-human primate-associated Staphylococcus schweitzeri sp. nov. Int J Syst Evol Microbiol.

[2] Chen SY, Lee H, Teng SH, Wang XM, Lee TF, Huang YC (2018). Accurate differentiation of novel Staphylococcus argenteus from Staphylococcus aureus using MALDI-TOF MS. Future Microbiol.

[3] Becker K, Schaumburg F, Kearns A, Larsen AR, Lindsay JA, Skov RL (2019). Implications of identifying the recently defined members of the Staphylococcus aureus complex S. argenteus and S. schweitzeri: a position paper of members of the ESCMID Study Group for Staphylococci and Staphylococcal Diseases (ESGS). Clin Microbiol Infect.

[4] Chantratita N, Wikraiphat C, Tandhavanant S, Wongsuvan G, Ariyaprasert P, Suntornsut P et al (2016) Comparison of community-onset Staphylococcus argenteus and *Staphylococcus aureus* sepsis in Thailand: a prospective multicentre observational study. Clin Microbiol Infect 22(5):458.e11–9. 10.1016/j.cmi.2016.01.008. 10.1016/j.cmi.2016.01.008 PMC4898209 26806258

[5] Chen SY, Lee H, Wang XM, Lee TF, Liao CH, Teng LJ (2018). High mortality impact of Staphylococcus argenteus on patients with community-onset staphylococcal bacteraemia. Int J Antimicrob Agents.

[6] Thaipadungpanit J, Amornchai P, Nickerson EK, Wongsuvan G, Wuthiekanun V, Limmathurotsakul D et al (2015) Clinical and molecular epidemiology of Staphylococcus argenteus infections in Thailand. J Clin Microbiol 53(3):1005–8. 10.1128/jcm.03049-14 10.1128/JCM.03049-14 PMC4390622 25568440

[7] Tong SY, Sharma-Kuinkel BK, Thaden JT, Whitney AR, Yang SJ, Mishra NN et al (2013) Virulence of endemic nonpigmented northern Australian *Staphylococcus aureus* clone (clonal complex 75, *S. argenteus*) is not augmented by staphyloxanthin. J Infect Dis 208(3):520–7 10.1093/infdis/jit173 10.1093/infdis/jit173 PMC3699000 23599317

[8] Argudin MA, Dodemont M, Vandendriessche S, Rottiers S, Tribes C, Roisin S (2016). Low occurrence of the new species Staphylococcus argenteus in a Staphylococcus aureus collection of human isolates from Belgium. Eur J Clin Microbiol Infect Dis.

[9] Enright MC, Day NP, Davies CE, Peacock SJ, Spratt BG (2000). Multilocus sequence typing for characterization of methicillin-resistant and methicillin-susceptible clones of *Staphylococcus aureus*. J Clin Microbiol.

[10] Ruimy R, Armand-Lefevre L, Barbier F, Ruppe E, Cocojaru R, Mesli Y (2009). Comparisons between geographically diverse samples of carried *Staphylococcus aureus*. J Bacteriol.

[11] Tang Hallback E, Karami N, Adlerberth I, Cardew S, Ohlen M, Engstrom Jakobsson H et al (2018) Methicillin-resistant Staphylococcus argenteus misidentified as methicillin-resistant *Staphylococcus aureus* emerging in western Sweden. J Med Microbiol. 10.1099/jmm.0.000760 10.1099/jmm.0.000760 29771232

